# Benzophenone-2 Concentration and Its Effect on Oxidative Stress and Apoptosis Markers in Rat Brain

**DOI:** 10.1007/s12640-019-0011-y

**Published:** 2019-04-22

**Authors:** Żaneta Broniowska, Beata Bystrowska, Beata Starek-Świechowicz, Bartosz Pomierny, Weronika Krzyżanowska, Maria Walczak, Bogusława Budziszewska

**Affiliations:** 1 Department of Biochemical Toxicology Medical College, Jagiellonian University, Medyczna 9, 30-688 Kraków, PL Poland; 2 Department of Toxicology, Chair of Toxicology, Medical College, Jagiellonian University, Medyczna 9, 30-688 Kraków, PL Poland

**Keywords:** UV filters, Benzophenone-2, Brain concentration, Oxidative stress

## Abstract

Benzophenones, frequently used as UV chemical filters, are absorbed through the skin and can exert systemic adverse effects. So far, most of the data are related to their action on sex hormone receptors whereas potential neurotoxic effect is expected mainly on the basis of in vitro studies. The aim of the present study was to determine concentrations of BP-2, oxidative stress and apoptosis markers in the rat brain after topical administration of this compound. Male Wistar rats were treated dermally with BP-2 (100 mg/kg, 4 weeks), and next, blood and tissue BP-2 concentrations and oxidative stress and apoptotic markers in the frontal cortex and hippocampus were determined. After dermal BP-2 administration, blood level of this compound was about 300 ng/ml while in the liver and adipose tissue 1354 and 823 ng/g wt tissue, respectively. In the studied brain structures, the levels of the test compound were from 5 to 19 ng/g tissue. In the hippocampus, where BP-2 level was about 3.5-fold lower than in the frontal cortex, no significant changes in either oxidative stress and apoptosis markers were observed. There was also no change in apoptosis markers in the frontal cortex but unexpectedly the oxidative stress markers were reduced. The research showed that BP-2 passes through the blood-brain barrier but its concentration in the brain structures are much lower than in the blood. This compound did not exacerbate oxidative stress and apoptosis markers in the hippocampus and frontal cortex, and even lowered oxidative stress in the frontal cortex.

## Introduction

Benzophenone derivatives are most common ultraviolet (UV) light filters used in a variety of cosmetics, like lipstics, shampoos, body lotions, conditioners, and hair sprays. They are also added to the production of food packaging to protect it from the damaging effects of sunlight. Negative effects of UV radiation, such as generation of reactive oxygen species (ROS), induction of DNA damage, and inhibition of skin immune system function increases the risk of skin cancer development, so the use of UV filters is required for protection of the skin. However, the existing information on potential adverse effects of chemical UV filters is incomplete and insufficient to reliably assess which of the currently used compounds are safest.

Human exposure to UV filters occurs mainly through skin absorption but also by consuming contaminated food (Jiang et al. 1999; Gustavsson Gonzalez et al. 2002). Due to widespread use of benzophenones, their high concentrations are detected in the waters of lakes and rivers as well as in wastewater and tissues of aquatic organisms, especially in fish fat (Fent et al. 2010). For many years, it was believed that the compounds used as chemical UV filters might only have adverse effects on the skin, i.e., might cause irritation or allergic contact dermatitis. However, subsequent studies showed that significant amounts of these compounds were absorbed by the skin, entered the circulation, and could exert systemic effects, mainly on the endocrine system function. Benzophenones belong to the group of endocrine-disrupting chemical (EDCs) because they show an affinity for the steroid hormone receptors and interfere with gonadal function. Most of the in vitro and in vivo data indicate estrogenic and antiandrogenic effects of these compounds, and some reports show that benzophenones also interfere with thyroid function. Estrogenic effect of BP-2 on the uterine weight, luteinizing hormone, and metabolic markers was demonstrated in ovariectomized rats (Jarry et al. 2004; Seidlová-Wuttke et al. 2004, 2005; Schlecht et al. 2006). Particularly unfavorable effects, like other EDCs, benzophenone-2 seems to exert on the development and function of testes. It has been found that human urinary concentration of benzophenone-2 is associated with decrease in semen quality and fertility (Buck Louis et al. 2015) and that administration of BP-2 to pregnant mice induce hypospadias in the male offspring and this effect is mediated through the estrogen receptors (Hsieh et al. 2007). In addition to the estrogenic effect, the administration of the BP-2 affects thyroid hormone secretion probably through inhibition of the thyroid peroxidase (TPO) activity (Schmutzler et al. 2007).

Benzophenones disrupting the function of sex and thyroid hormones may affect almost all systems of the body, because estrogen and thyroid receptors occur in many cells. Central nervous system cells also contain receptors for the sex and thyroid hormones, so benzophenones can impair their function. The role of estrogens in regulating the function of nerve cells, especially their neuroprotective effects, is well known (Engler-Chiurazzi et al. 2017). Thyroid hormones are essential for the proper functioning of the brain primarily during the developmental period but current research indicates that also in adults, some genes, especially those involved in regulating brain metabolism and intracellular signal pathways, remain sensitive to the action of these hormones (Vallortigara et al. 2008; Wang et al. 2015b). Thus, benzophenones impairing the sex and the thyroid hormone function in the brain can affect viability of the CNS cells and thus exacerbate neurodegenerative processes. This possibility is confirmed by results of in vitro studies. Previously, we showed that various chemical UV filters, including BP-2, decreased viability and increased the process of apoptosis in SH-SY5Y human neuroblastoma cells (Broniowska et al. 2016). Although, BP-2 exerted necrotic effect in relatively high concentration of about 10 μM; however, the pro-apoptotic action of this compound was already evident when it was used in much lower concentrations of about 0.1 μM, so concentrations that theoretically may be present in vivo. Taking into account the lipophilic nature of benzophenones, they should pass through the blood-brain barrier, but up to now, the level of any of the benzophenones in the central nervous system in in vivo models is not known. As far as BP-2 is concerned, there is even no data about its absorption through the skin and blood levels after dermal application. Generally, it is estimated that about 10% of the dermal dose of benzophenones is absorbed into the systemic circulation (Jiang et al. 1999; Gustavsson Gonzalez et al. 2002). Among benzophenones, the most data concerns absorption and blood level of benzophenone 3 in humans and experimental animals. After dermal application, benzophenone 3 concentration in human blood is up to 250–300 ng/ml and it has been shown that in the liver, kidneys, spleen, and testes of rats, the level of this compound can be up to 10-fold higher than in serum (Okereke et al. 1994; Tarazona et al. 2013). Since there are no data on the levels of benzophenone 2 in the body after its dermal administration, the first aim of the current research was to determine its level in blood, peripheral tissues (liver, adipose tissue), and in brain structures (frontal cortex, hippocampus, cerebellum) after 4-week topical administration. The liver was chosen for the study because it is responsible for metabolism of xenobiotics, and, therefore, usually, they are present in high concentrations in this organ. Also in adipose tissue, due to the lipophilic nature of BP-2 and its possible accumulation, we expected to find relatively high levels of this compound (Wang et al. 2015a). Among brain structures, the cerebellum was chosen, as a tissue less sensitive to the neurotoxic effects of xenobiotics and frontal cortex and hippocampus, because they are most vulnerable to damage. The second objective of the present study was to demonstrate whether benzophenone-2 administered dermally for 4 weeks induces changes, which evidence damage to the tissues in the most vulnerable brain structures, i.e., in the frontal cortex and hippocampus. Since the main cause of neuronal injury is oxidative stress and a reduction in concentrations of already low brain levels of antioxidant enzymes, therefore, to evaluate a potential adverse BP-2 effect on brain structures, the production of reactive oxygen species (ROS), total antioxidant activity, and lipid peroxidation were assayed. Oxidative stress is the main cause of the induction of apoptosis which plays the main role in pathogenesis of neurodegenerative disorders (Radi et al. 2014). Therefore, in order to demonstrate whether the excessive exposure to BP-2 may be involved in induction or exacerbation of neurodegenerative changes, we also investigated the effect of this compound on apoptotic markers, such as active form of caspase-3, pro-apoptotic protein (Bax), and anti-apoptotic protein (Bcl-2).

## Materials and Methods

### Materials

Benzophenone-2 (2,2′,4,4′-tetrahydroxybenzofenone CAS No 131-55-5) was purchased from Sigma Chemical Co. (St. Louis, MO).

### Animal Treatment

The experiments were performed on male Wistar rats (225–250 g) delivered from the animal house facility of the Jagiellonian University Medical College in Cracow. The animals were kept under natural day-night cycle, at 22 ± 2 °C with food and water available at libitum. All procedures were conducted according to the NIH Guide for the Care and Use of Laboratory Animals and were approved by the local ethics committee. The rats were randomly divided into two groups of ten animals in each; the first group was treated with gel with BP-2 and the second group with pure gel.

Hair on the back of the neck through half-way towards the tail region was shaved off prior to treatment. Animals were reshaved during the course of treatment as soon as the hair began to reappear. BP-2 was dissolved in small amount of ethanol and olive oil and formulated with Hascobase (Hasco-Lek, Poland) to the ointment and was applied at a dose 100 mg/kg twice a day (8:00 and 17:00) for 4 weeks. Control animals were administered Hascobase with a small amount of ethanol and olive oil. Prior to each application of BP-2, the skin was cleaned with gauze soaked in distilled water. The chemical was then applied gently and spread thinly over the shaved areas of the skin. Body weights of rats were recorded on weekly basis and on the day of sacrifice.

BP-2 or Hascobase without this compound was applied for 4 weeks twice a day, and the animals were killed 24 h after the last dose. The animals were observed daily for any clinical signs of impairment of health.

### Tissue Collection

Twenty-four hours after last BP-2 administration, animals were killed under non-stress conditions by rapid decapitation and blood was collected in anticoagulant tubes. Brain regions (hippocampus, frontal cortex, and cerebellum) and peripheral tissues (liver, adipose tissue) were dissected on ice-cold glass plates and stored at − 80 °C until they were used for biochemical assays.

### LC/MS Analysis

LC/MS-grade methanol, heptane, ethyl acetate were obtained from Sigma-Aldrich (St. Louis, USA). LC/MS-grade glacial acetic acid was obtained from Chempur (Piekary Śląskie, Poland). Benzophenone-2 was purchased from Sigma Chemical Co. (St. Louis, MO). Benzophenone-d10 from Sigma-Aldrich (St. Louis, USA) was used as an internal standard (IS) with isotope labeling. A working internal standard solution of BP-d10 was prepared at a concentration 25 μg/ml in LC/MS grade methanol. All standards were stored at − 20 °C in the dark.

Frozen tissues were weighed, thawed at room temperature, and homogenized in water in proportion 10 mg tissue (liver, adipose tissue) or 20 mg tissue (frontal cortex, hippocampus, cerebellum) per 200 μl of water. Plasma was mixed with water in proportion 1:1. Next, 200 μl of homogenate or diluted plasma was mixed with 2 μl of internal standard in methanol and 200 μl of water. A 1.5-ml mixture heptane and ethyl acetate (1:1; *v*/*v*) were added, and the samples were shaken for 10 min on an oscillating shaker. The samples were then centrifuged for 10 min at 4000×*g* and the organic layer was collected. The organic phase was evaporated to dryness under a stream of nitrogen at 37 °C. The residues were reconstituted in 100 μl of methanol and subjected to chromatographic analysis.

Additionally, to assess the level of total BP-2 (parent compound and its metabolites—glucuronide and sulfate) liver was homogenized in 1 M ammonium acetate buffer, pH 5.0 in proportion 10 mg tissue per 200 μl of buffer and plasma was mixed with 1 M ammonium acetate buffer in proportion 1:1. Immediately prior to the incubation for deconjugation to all samples, 10 μl of freshly prepared enzyme mixtures (20 *v*/*v* % β-glucuronidase and 20 *v*/*v* % sulfatase dissolved in 1 M ammonium acetate buffer, pH 5.0) for 200 μl of homogenate were added. The samples were mixed and incubated for 6 h in water bath. The enzyme reaction was terminated by frozen in − 80 °C.

The chromatographic separation was performed on an Agilent HPLC 1100 series system (Agilent, Waldbronn, Germany), which was equipped with a degasser, a binary pump, an auto-sampler, and a thermostated column compartment, as we described previously (Smaga et al. 2017). The tissue sample was separated on a Xterra RP18 (Waters) column (100 mm × 3.0 mm ID, 3.5 μm particle size). The column was thermostated at 30 °C. The mobile phase was composed of 0.25% glacial acetic acid in water (B) and methanol (A) using the following gradient program: 0–1.5 min, isocratic gradient 40.0% (A); 1.5–2.5 min, linear gradient 40.0–95.0% (A); 2.50–6.50 min, isocratic gradient 95.0% (A); 6.5–8.0 min, linear gradient 95.0–40.0% (A); 8.0–10.0 min, isocratic gradient 40.0% (A); the flow rate was 0.4 mL/min; the injection volume was 40 μL.

Mass spectrometric analyses were accomplished on an Applied Biosystems MDS Sciex (Concord, Ontario, Canada) API 2000 triple quadrupole mass spectrometer equipped with an electrospray ionization (ESI) interface. ESI ionization was performed in the positive ionization mode. High-purity nitrogen used as a sheath gas was generated with a nitrogen generator. All experiments were carried out in the positive ion mode. The ion source parameters were as follows: ion spray voltage (IS) 5000 V; nebulizer gas (gas 1) 20 psi; turbo gas (gas 2) 10 psi; temperature of the heated nebulizer (TEM) 250 °C; curtain gas (CUR) 20 psi. Nitrogen (99.9%) from Peak NM20ZA was used as the curtain and collision gas. The ion path parameters for BP-2 and BP-d10 were as follows: declustering potential (DP) 8 V; focusing potential (FP) 380 V; entrance potential (EP) 10 V; collision cell entrance potential (CEP) 13 V; collision cell exit potential (CXP) 18 V, respectively. The quantization analysis was performed using the MRM mode and tandem LC/MS. The following pairs of ions were monitored with the following values of m/z: 247.0/137.1 for BP-2 and 193.0/110.0 for BP-d10. Data were analyzed by using the Analyst software 1.6 (Perlan Technologies). The levels of benzophenone-2 were calculated using the calibration standard curves, constructed by linear regression analysis of peak area versus concentration curves.

### ROS Assay

Brain structures were homogenized in an ice-cold 0.1 M phosphate-buffered solution (PBS, pH 7.4). The homogenates were centrifuged at 13,000 rpm for 20 min at 4 °C. The supernatants were maintained at − 80 °C until being used for biochemical analysis.

Briefly, the fluorescence dye DCFH-DA was de-acetylated by cellular esterases into a non-fluorescent compound that could be oxidized by ROS into 2′,7′-dichlorofulorescein (DCF). To determine the influence of BP-2 on reactive oxygen species (ROS) production, supernatants of brain structures were transferred with 10 μM solution of 2′,7′-dichlorodihydrofluorescein (DCFH-DA) diacetate to black 96-well plates. DCFH-DA solution was co-incubated at 37 °C for 20 min, than cooled in an ice-bath for 15 min. DCF fluorescence was measured using a fluorescence plate reader (Fluoroskan Ascent FL, Thermo Labsystems) at 485 nm excitation and 535 nm emission wavelengths. ROS production in samples was calculated from the standard curve and was expressed as nmol of ROS per mg of protein.

### Lipid Peroxidation (MDA) Level

Fluorometric assay for lipid peroxidation was performed using lipid peroxidation (MDA) colorimetric/fluorometric assay kit (BioVision). The assay is based on the reaction of the main product of the lipid peroxidation-malondialdehyde (MDA) with thiobarbituric acid (TBA) at 95 °C for 60 min. The product of this reaction MDA-TBA adduct can be quantified colorimetrically (OD 532 nm) or fluorometrically (Ex/Em = 532/553). The fluorescence was measured by a fluorescence plate reader (Fluoroskan Ascent FL; Thermo Labsystems). Lipid peroxidation in samples was calculated from the standard curve and expressed as nmol of MDA/mg of protein.

### Total Antioxidant Capacity

Analysis of total antioxidant capacity (TAC) was carried out on the brain structure homogenates according to the modified method of Benzie and Strain (1996) adapted to specific tissue and microplate assay. The tissue antioxidant capacity was evaluated by reduction of ferric ions. Fe(III)–tripyridyltriazine (Fe(III)–TPTZ) complex was reduced to blue Fe(II)–tripyridyltriazine (Fe(II)–TPTZ), the level of which was determined spectrophotometrically at 573 nm. The absorbance was measured by a multiwell plate reader (TECAN Infinite M200 PRO). The total antioxidant capacity of samples was calculated from the standard curve and expressed as μmol Fe(II) per mg of protein.

### Determination of Active Form of Caspase-3, Pro-Apoptotic Protein (Bax), and Anti-Apoptotic Protein (Bcl-2)

Apoptotic markers were determined by Western blot method, as we described previously (Pomierny et al. 2016). Fragments of the frontal cortex and hippocampal tissues were homogenized in 2% SDS containing 1 mM PMSF, 1 mM Na_2_VO_4_, 20 mM NaF, and mixture of phosphatase and proteinase inhibitors (Sigma-Aldrich) using Ultra-Turrax and ultrasonic homogenizers. Homogenates were then denatured for 10 min at 95 °C, and insoluble material was discarded by centrifugation at 10,000 rpm for 10 min at 4 °C. Ten microliters of supernatants were used for protein determination and after adjusting to a proper protein concentration, solutions were mixed with loading buffer (containing 10% 2-mercaptoethanol) at the ratio of 1:1 and heated at 95 °C for 10 min. Samples were stored at − 80 °C. Protein samples were loaded on the gradient 8–16% SDS polyacrylamide gels (Bio Rad Corp.) at the concentration of 30 μg/10 μl and electrophoreses (140 V, 1 h) were performed. Proteins were transferred to PVDF membranes on ice at 110 V for 1 h. Membranes were then blocked in 5% nonfat milk prepared in TBST. Particular membranes were incubated overnight at 4 °C with solutions of primary antibodies in 1% nonfat milk against: Bcl-2 and Bax (Santa Cruz Biotechnology, mouse monoclonal antibodies at the concentration of 1:500) and active form of caspase-3 (Santa Cruz Biotechnology, rabbit polyclonal antibody at the concentration of 1:700). After overnight incubation membranes were washed twice in TBST, twice in 1% nonfat milk, and incubated with 1% nonfat milk solution of respective secondary antibodies conjugated with peroxidase-anti-mouse (1:5000, Santa Cruz Biotechnology) or anti-rabbit (1:5000, Santa Cruz Biotechnology) for 1 h in the room temperature. After washing in TBST, membranes were developed using ECL method (Western Bright Quantum, Advansta Inc., USA), and the membrane chemiluminescence was imaged with G-Box Imaging System (Syngene, USA). The expression of particular proteins was analyzed with Gene Tools software (v. 4.03, Syngene, USA) and expressed as relative to total protein (staining with Pierce™ Reversible Protein Stain Kit for PVDF Membranes, Thermo Fisher Scientific).

### Brain Protein Concentration

Protein concentration in the brain structure homogenates was determined using bicinchoninic acid. This method combines reduction of Cu^+2^ to Cu^+1^ by protein in an alkaline medium (the biuret reaction) with the colorimetric detection of the cuprous cation (Cu^+1^) using the bicinchoninic acid. The purple-colored reaction product of this assay is formed by the chelation of two molecules of BCA with one cuprous ion. This water-soluble complex exhibits a strong absorbance at 562 nm, which is linear with increasing protein concentrations. The absorbance was measured by a multiwell plate reader TECAN Infinite M200.

### Statistical Analysis

All data were expressed as the means (±SEM) from ten samples assayed in duplicates. Statistical analyses were performed with a one-way analysis of variance (ANOVA) and next, differences between groups were evaluated by the Dunnett’s post hoc test. *p* < 0.05 was considered as statistically significant.

## Results

A 4-week twice a day, dermal administration of benzophenone-2 at a dose of 100 mg/kg did not significantly alter the body weight or cause any apparent adverse effects, compared to control animals receiving only the base.

### Concentration of Benzophenone-2 in Plasma, Peripheral Tissues, and Brain Structures

**Fig. 1:**
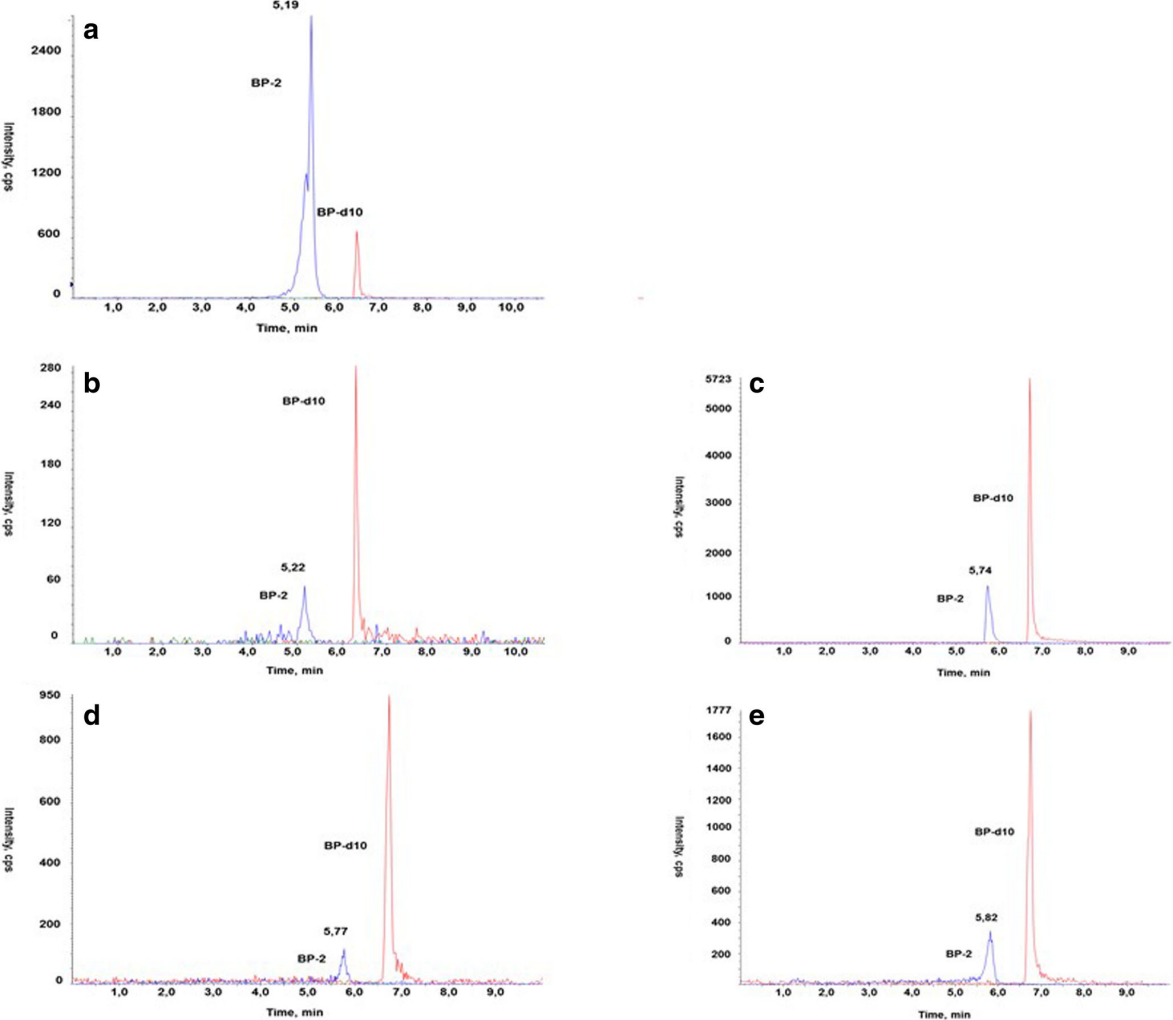
A representative chromatogram of BP-2 standard (**a**) and serum of BP-2 treated rats without (**b**) and after (**c**) glucuronide and sulfate hydrolysis, and liver without (**d**) and after (**e**) hydrolysis

Based on chromatograms of reference standards, the peak with the retention time of 5.4–5.8 min was identified as BP-2 (Fig. 1a). A molar mass of this peak was 246 g/mol, and this value corresponds to the molar mass of the BP-2. The LC/MS chromatograms of plasma, liver, adipose tissue, frontal cortex, hippocampus, and cerebellum from BP-2-treated animals showed a single peak with the retention time about 5.8 min, which was not detectable in samples from control animals. Representative chromatograms of serum and liver sample from control and BP-2-treated animals are shown in Fig. 1b, d. After hydrolysis with β-glucuronidase and sulfatase, the BP-2 peak was significantly higher than in the same serum and liver samples before hydrolysis (Fig. 1c, e).

**Fig. 2:**
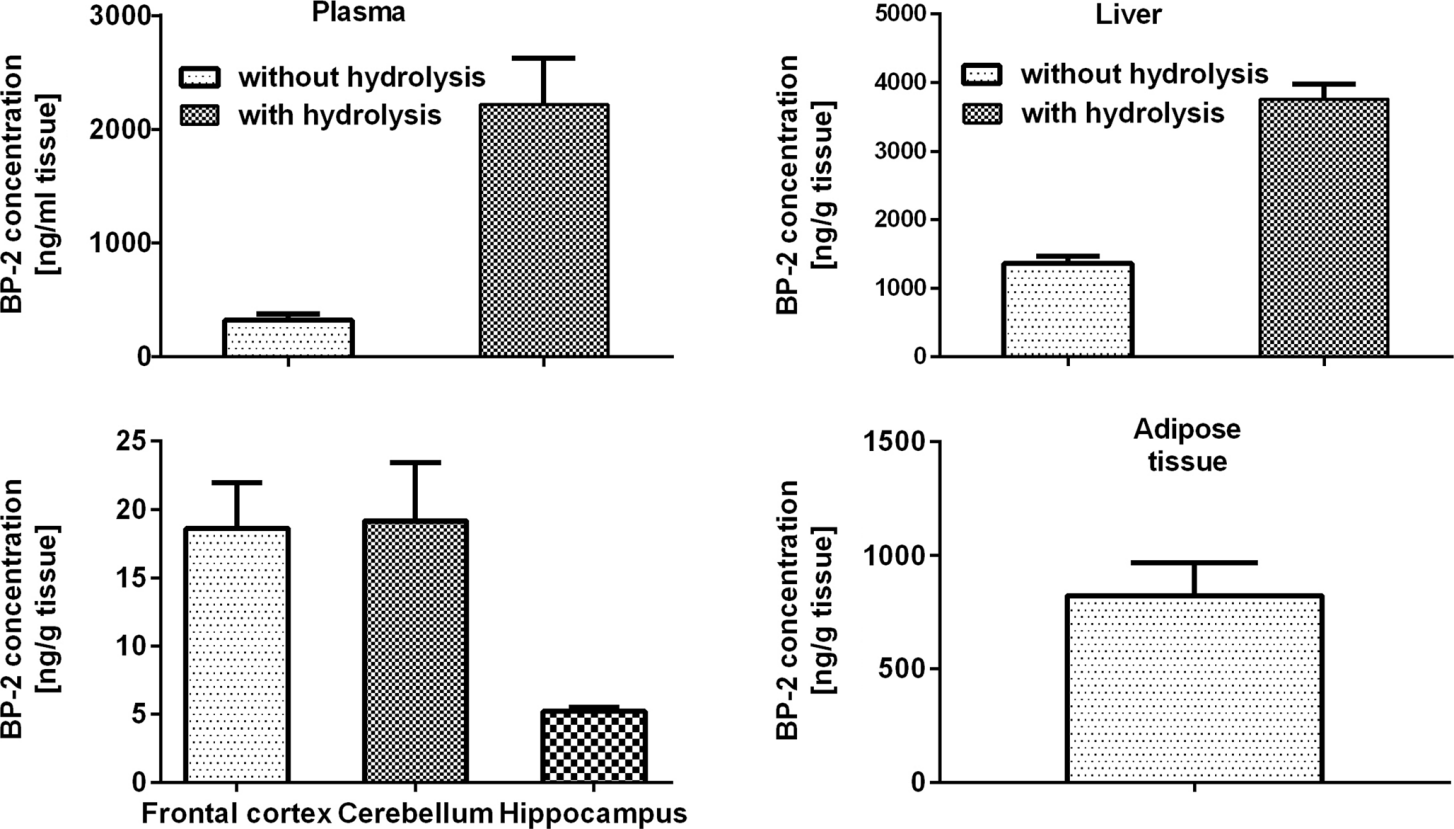
Concentrations of free BP-2 in serum, liver, adipose tissue, cerebellum, frontal cortex, and hippocampus and total BP-2 concentrations after glucuronide and sulfate hydrolysis in serum and liver. Rats were treated dermally with 100 mg/kg BP-2 twice a day for 4 weeks and were killed 24 h after the last BP-2 application. Data are expressed as the means ± SEM and were statistically evaluated by one-way analysis of variance (ANOVA), followed by Duncan’s post hoc test. *p < 0.05 vs. control animals, *n* = 10

Calculation of benzophenone-2 concentration from the calibration standard curves revealed that the test compound was present in the plasma of treated animals in a concentration range from 164 to 648 ng/ml (the average 324 ng/ml; 1.3 μM) (Fig. 2). After hydrolysis, the concentration of this compound was 2218 ng/ml (9 μM), which indicated that in blood, there was more BP-2 metabolites than the parent compound (Fig. 2). Also in the liver, the BP-2 concentration after hydrolysis was much higher (3758 ng/g) than the free form of this compound (1482 ng/g) (Fig. 2), although in this tissue, the free form was ca. 40% of the total form while in serum, only about 15% of the total BP-2 occurred in unmetabolized form. Since the biological effect is induced by free BP-2, in other tissues, only this form was determined. It was found that the average concentration of this compound in adipose tissue was 823 ng/g; in the cerebellum and frontal cortex, it was 19 ng/g; and in the hippocampus 5 ng/g (Fig. 2).

The present study showed that after the dermal 4-week BP-2 administration, this compound in the examined peripheral tissues was present at micromolar concentrations (5.5 μM in the liver, 3.3 μM in adipose tissue), whereas in the brain structures at much lower concentrations (0.08 μM in the cerebellum and frontal cortex and 0.02 μM in the hippocampus). BP-2 concentrations in all examined tissues in control animals were below the detection limit, but in animals receiving this compound, BP-2 was present in plasma, liver, and adipose tissue in all animals, whereas among brain structures, this compound was below the detection limit in the cerebellum and frontal cortex in one rat and in the hippocampus in two animals.

### The Effect of BP-2 on Reactive Oxygen Species, Total Antioxidant Capacity, and Lipid Peroxidation

**Fig. 3:**
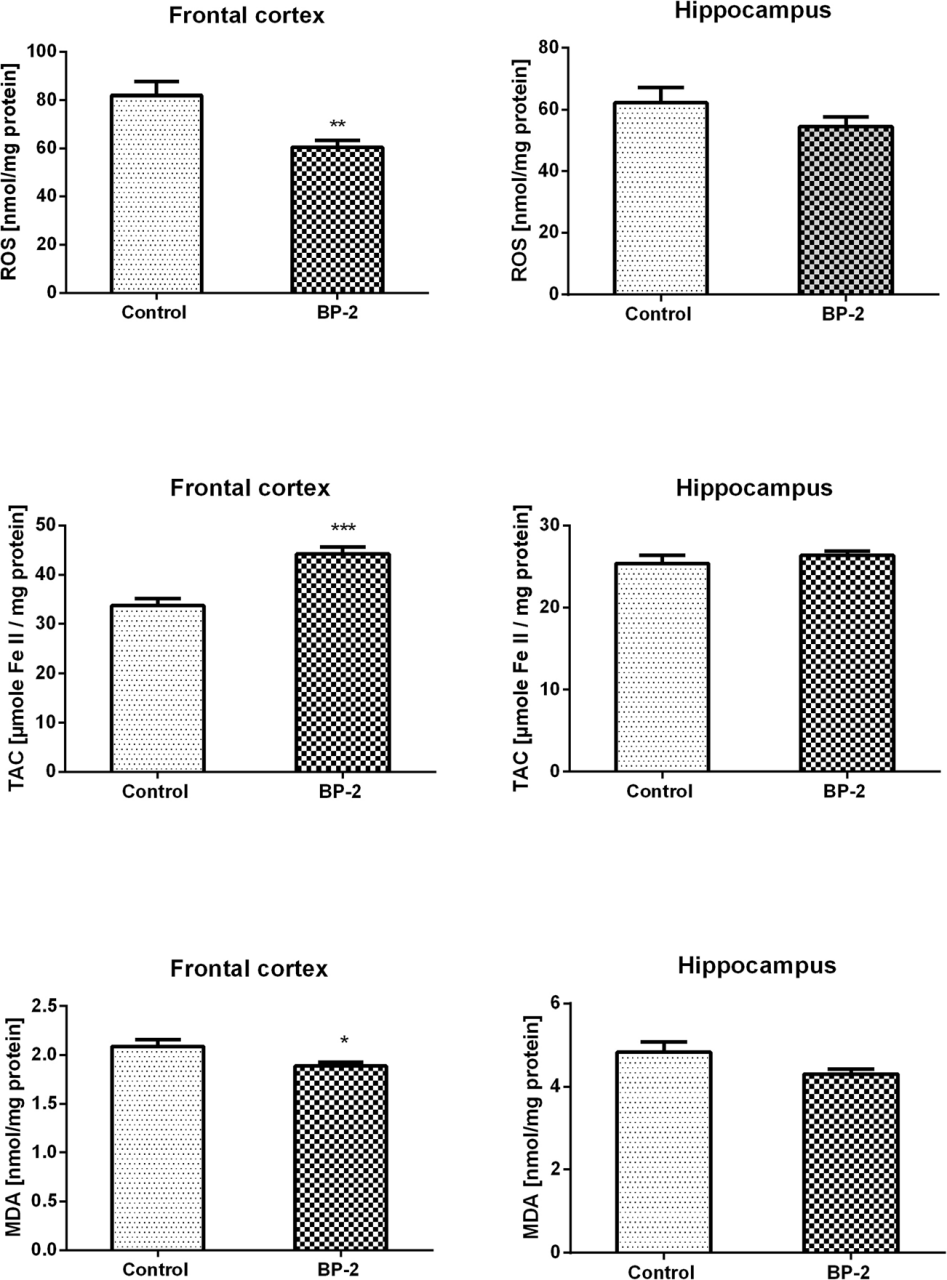
The effect of BP-2 on reactive oxygen species (ROS) level in the frontal cortex and hippocampus, on total antioxidant capacity (TAC) in the frontal cortex and hippocampus and lipid peroxidation in the frontal cortex and hippocampus. Rats were treated dermally with 100 mg/kg BP-2 twice a day for 4 weeks and were killed 24 h after the last BP-2 application. Data are expressed as the means ± SEM and were statistically evaluated by one-way analysis of variance (ANOVA), followed by Duncan’s post hoc test. *p < 0.05 vs. control animals, *n* = 10

To determine whether BP-2 influences the oxidative stress parameters in the most sensitive brain structures, the level of reactive oxygen species (ROS), total antioxidant capacity (TAC), and lipid peroxidation were determined in the hippocampus and frontal cortex. In control animals, the levels of ROS and TAC were slightly higher in the frontal cortex than in the hippocampus, whereas concentration of MDA, a product of lipid peroxidation was about 2-fold higher in the hippocampus than in the frontal cortex. It was found that BP-2 administration did not significantly affect any of the examined oxidative stress markers in the hippocampus (Fig. 3) and even a tendency to decrease the level of ROS and lipid peroxidation was observed in BP-2-treated rats in comparison to control animals. In the second of the examined brain structures, frontal cortex, a statistically significant decrease in ROS production (about 26%) and increase of total antioxidant activity (about 31%) was demonstrated (Fig. 3). The level of the main polyunsaturated fatty acid peroxidation product malondialdehyde (MDA) was significantly lower (about 10%) in the frontal cortex of BP-2-treated rats than in control animals (Fig. 3).

### The Effect of BP-2 on Apoptotic Markers

To determine whether BP-2 can affect process of apoptosis, the active form of caspase-3 (17 kDa fragment created by cleavage of caspase-3), main anti-apoptotic (Bcl-2), and primary pro-apoptotic (Bax) proteins were determined by Western blot method in the hippocampus and frontal cortex in BP-2-treated and control animals. We have shown that BP-2 administration did not change in statistically significant manner the concentrations of any of the examined apoptotic markers, neither in the hippocampus nor in the frontal cortex (Table 1).

**Table 1:** The effect of benzophenone-2 (BP-2) on expression of active form of caspase-3, pro-apoptotic (Bax), and anti-apoptotic (Bcl-2) proteins in the frontal cortex and hippocampus. Rats were treated dermally with 100 mg/kg BP-2 twice a day for 4 weeks and were killed 24 h after the last BP-2 application. Data are expressed as the means ± SEM and were statistically evaluated by one-way analysis of variance (ANOVA), followed by Duncan’s post hoc test (*n* = 10)

	Control	BP-2
	Frontal cortex	
Caspase-3 active	100 ± 10.22	92.1 ± 11.51
Bax	100 ± 7.1	107.7 ± 7.5
Bcl-2	100 ± 5.51	103.2 ± 8,56
	Hippocampus	
Caspase-3 active	100 ± 11.41	112.2 ± 7.62
Bax	100 ± 7.62	88.22 ± 4.48
Bcl-2	100 ± 8.65	102.6 ± 11.25

## Discussion

The present study demonstrates that BP-2 is absorbed through the skin and occurs in micromolar concentrations in the blood, liver, and adipose tissue. We provide also the evidence that this compound passes through the blood-brain barrier and is present in varying, but relatively low, concentrations in the certain brain structures. The obtained results also show that after 4 weeks of dermal exposure of rats to BP-2, the concentration of this compound in the brain is too low to induce oxidative stress or induce apoptosis in the hippocampus and frontal cortex.

To the best of our knowledge, this is the first study in which concentrations of BP-2 in plasma, peripheral tissues, and brain structures were determined after dermal sub-chronic application of this compound. To date, only serum BP-2 concentration was determined in rats after a short, 5-day administration of this compound per gavage (Schlecht et al. 2008). We found that 24-h after last, dermal BP-2 application, plasma concentration of this compound was 324 ng/ml, whereas after hydrolysis, total level of this compound was ca. 7-fold higher than free form of BP-2. These results are consistent with data obtained in rats after oral administration of this compound, which showed that in serum and urine, BP-2 metabolites were present at much higher concentration than free BP-2 (Schlecht et al. 2008). The existing data on the BP-2 metabolism are scarce, but it was stated that in zebrafish, BP-2 was exclusively metabolized through phase II, but not by phase I reaction (Le Fol et al. 2017). Research described by Schlecht et al. (2008) indicate that also in the rat, BP-2 is metabolized only via phase II, and specifically, after oral administration, this compound is rapidly conjugated mainly with glucuronate and to a smaller extent with sulfate and excreted with urine. We found that more BP-2 in the form of conjugates occurred in the serum than in the liver, the main organ involved in metabolism of xenobiotics, which suggests that the metabolism of this compound also occurs in other organs. The gut can be another probable organ of BP-2 metabolism, because synthetic steroids, for which benzophenones show structural similarity, were reported to be metabolized mainly in the gut (Back et al. 1990). The relatively high concentration of BP-2 occurred in adipose tissue, indicating that, like other lipophilic xenobiotics, this compound is accumulated in this tissue.

The obtained data showed that 24 h after the last dermal administration of BP-2, its concentrations in blood and peripheral tissues, such as liver and adipose tissue, were relatively high. As for serum, similar BP-2 levels as in the present study have been demonstrated after oral administration of this compound, but only for a short time after administration. After oral administration, the maximum BP-2 concentration in serum was observed after 30 min and depending on the dose, it ranged from 0.1 to 1.1 μg/ml (Schlecht et al. 2008), whereas in the case of dermal administration, the BP-2 level of about 0.3 μg/ml was observed 24 h after the last injection, suggesting a slower metabolism of the test compound after dermal than oral administration. High concentrations of BP-2 were observed in the liver, which is presumably due to the fact that this compound is metabolized in this organ and in adipose tissue, which is more likely connected with accumulation (Wang et al. 2015a). Many studies conducted on fish indicate that benzophenones are cumulated mainly in this tissue. As a lipophilic compound, BP-2 is not only accumulated in adipose tissue but also should cross blood-brain barrier. The present study provided evidence that it actually reached the brain tissue, but it was found in brain structures, especially in the hippocampus, rather at low concentrations. Because the BP-2 level was measured only at one time point, after a relatively long time after its administration, it can be assumed that in earlier time points, its concentration might be higher. Of the brain structures most susceptible to damage, higher levels of BP-2 occurred in the frontal cortex than in the hippocampus, which is consistent with the often observed phenomenon of greater distribution of lipophilic xenobiotics in the cerebral cortex than in subcortical areas.

The obtained results showed that after 2-week dermal administration of BP-2, the concentrations of this compound in the examined tissues were different. The highest concentration was observed in the liver, which is well-perfused and possessing a strong metabolic capacity tissue. In contrast to the liver, the adipose tissue and brain have a low metabolic phase II capacity and in these tissues, BP-2, like other EDC with similar chemical structure, should be present mainly in free but not conjugated form (Geens et al. 2012). Despite the fact that BP-2 is a lipophilic compound, its concentration in the adipose tissue was a little lower and in the brain much lower than the level of the free form of this compound in more hydrophilic organs, especially in the liver. The unexpectedly low BP-2 concentration in the brain is difficult to explain, but it may be due to its limited ability to pass through the blood-brain barrier or the rapid removal of this compound by transporters present in the blood-brain barrier. BP-2 is a less lipophilic compound than benzophenone-3 (BP-3), which as shown by our unpublished data, reaches in the rat brain approximately 2.5-fold higher concentration than the BP-2. Differences in octane/water partition coefficients may account for differences in brain concentrations between BP-3 (log Kow about 3.6) and BP-2 (log Kow about 3.2); however, the relatively low concentrations of both BP-2 and BP-3 in the rat brain suggest that these compounds could be removed by some blood-brain barrier transporters. As in the case of BP-2, also another EDC-bisphenol-A, with a structure similar to benzophenones poorly penetrates into the brain, which also suggests that, unlike endogenous estrogens, transport to the brain of xenobiotics showing affinity for estrogen receptors may be limited (Sun et al. 2002). Like the blood-brain barrier, also the placental barrier seems to give some protection of the fetuses against certain benzophenones (Krause et al. 2018).

In the second part of this study, we determined potential adverse effects of BP-2 on oxidative stress and apoptotic markers in the frontal cortex and hippocampus, which are the structures especially vulnerable to damage. Contrary to predictions, in none of these brain regions, changes in oxidative stress and apoptotic markers were observed, which would indicate the induction of cell damage. We have found that 4-week dermal administration of BP-2 did not change the level of the active form of caspase-3, executive enzyme in apoptotic process, or key proteins regulating apoptosis in both brain regions. Thus, the obtained results did not confirm our previous data from in vitro study (Broniowska et al. 2016). In previous studies, we have shown that BP-2 present in culture medium for 72 h in concentrations range from 0.1 to 100 μM induces apoptosis in human neuroblastoma cell line—SH-SY5Y. In the case of the hippocampus, in this structure, BP-2 was present at a concentration of ca. 0.02 μM, so at a concentration lower than the concentrations that induced apoptosis in SH-SY5Y cells. However, in the frontal cortex, BP-2 concentration was only slightly lower (0.08 μM) from concentrations that induced apoptosis in SH-SY5Y cells. Interestingly, in the frontal cortex, not only no changes in the apoptosis markers were observed, but even BP-2 caused beneficial changes in oxidative stress markers. This compound reduced the level of ROS, enhanced total antioxidant capacity, and lowered lipid peroxidation, so to a certain extent, it augmented the endogenous mechanisms of neuroprotection. Induction of total antioxidant activity by BP-2 was probably the cause of inactivation and reduction in ROS level and in consequence decreasing lipid peroxidation. The increase in total antioxidant capacity may result from intensification of synthesis of endogenous antioxidant enzymes or non-enzymatic antioxidants. Unlike in the frontal cortex, in the hippocampus, there were no changes in the oxidative stress markers, which may be due to low BP-2 levels in this structure.

The beneficial effect of BP-2 in the frontal cortex is difficult to explain especially because previous data have shown that another compound from the benzophenone group, i.e., benzophenone-3 induced strong neurotoxic effects in this structure (Krzyżanowska et al. 2018; Wnuk et al. 2018). The difference in the effects of these compounds results probably from their penetration into the CSN and from their different chemical structures. BP-2 is less lipophilic than benzophenone-3 which may be the main reason for its poorer penetration to the brain and, therefore, lack of neurotoxic effect. On the other hand, it is known that even a slight change in the structure of the compound can significantly alter its biological effects. For example, examining the effect of different fluorinated benzophenone derivatives in in vitro models, it has been shown that depending on the chemical structure, some of them exhibit antioxidant properties (Belluti et al. 2014). Based on the existing data, it is not possible to determine whether the antioxidant effects of BP-2 in the frontal cortex result from the activation of endogenous, antioxidant mechanisms triggered by low concentrations of neurotoxic compounds or is the result of the antioxidant properties of this compound; however, since the BP-2 in vitro produces a distinct pro-apoptotic effect, the former suggestion is more likely.

In conclusion, the present study showed that BP-2 was absorbed through the rat skin and passed through the blood-brain barrier, but its concentration in the brain was much lower than in the periphery. In the animal model used, BP-2 did not show pro-oxidative or pro-apoptotic effects in the hippocampus and frontal cortex, and in the frontal cortex even reduced the parameters of oxidative stress.
